# Association between clinical biomechanical metrics of cervical spine function and pain or disability in people with neuromusculoskeletal neck pain: Protocol for a systematic review and planned meta-analysis

**DOI:** 10.1371/journal.pone.0303365

**Published:** 2024-05-10

**Authors:** Saghar Soltanabadi, Sima Vatandoost, Michael J. Lukacs, Alison Rushton, David M. Walton

**Affiliations:** 1 School of Physical Therapy, Western University, London, Ontario, Canada; 2 Physiotherapy Department, London Health Science Center (LHSC), London, Ontario, Canada; UFPE: Universidade Federal de Pernambuco, BRAZIL

## Abstract

**Introduction/Background:**

Neck pain is a burdensome condition associated with pain, disability, and economic cost. Neck pain has been associated with observable changes in neuromuscular function and biomechanics. Prior research shows impairments in kinematic control, including reduced mobility, velocity, and smoothness of cervical motion. However, the strength of association between these impairments and patient-reported pain and disability is unclear rendering development of novel and relevant rehabilitation strategies difficult.

The aim of this systematic review is to synthesize existing evidence on the strength of association between clinical biomechanical metrics of neck function (ROM, strength, acceleration, accuracy, smoothness, etc.) and patient-reported neck pain and disability.

**Methods/Analysis:**

This protocol follows Cochrane guidelines and adheres to the Preferred Reporting Items for Systematic Reviews and Meta-Analyses Protocols (PRISMA-P). MEDLINE, EMBASE, CINAHL, SPORTDiscus, Web of Science and Scopus will be searched, along with the gray literature, up to 20 November 2023, using terms and keywords derived from initial scoping searches. Observational studies, including cohorts and cross-sectional studies, that explore associations between clinical biomechanics of the neck and patient-reported outcomes of neck pain or disability will be included. Two reviewers will independently perform study selection, data extraction, and risk of bias assessment (National Institute of Health tool). Data will be synthesized using either a random effects meta-analytic approach or qualitatively using a modified Grading of Recommendations, Assessment, Development and Evaluation (GRADE) approach, dependent on the homogeneity of data available.

**Discussion and relevance:**

This review addresses a gap in the literature by systematically synthesizing findings on the relationship between neck function impairments and patient-reported outcomes. It will identify priorities for neck pain rehabilitation and gaps in current knowledge.

**Dissemination:**

The results of this review will be disseminated through a peer-reviewed publication, conference presentation, and lay language summaries posted on an open-access website.

**Trial registration:**

**PROSPERO Registration number:** CRD42023417317. https://www.crd.york.ac.uk/prospero/display_record.php?ID=CRD42023417317.

## Introduction

Neck pain (NP) is defined as pain primarily experienced in the region bound by the occiput cranially, 1^st^ thoracic vertebra caudally, and lateral points of the scapular spines laterally, with or without referred pain in one or both upper limbs that lasts for at least one day [1,2]. NP is a major global public health concern; recent literature has indicated an estimated global point prevalence of 7.6% and a lifetime prevalence of 48.5% in the adult population [3]. Furthermore, NP often recurs as episodes throughout the lifetime [4,5]. NP is associated with disability [6], psychological comorbidity [7,8], as well as significant healthcare and financial expenses due to missed work hours and reduced productivity [9]. The Global Burden of Diseases studies have ranked neck pain fourth in terms of overall disability and 21st in terms of overall burden amongst 291 conditions studied [1,2].

Over the past three decades, despite active research efforts, the age-standardized point prevalence, annual incidence, and rates of years lived with disability associated with neck pain (NP) have remained largely unchanged [1]. There is a clear need for effective methods to evaluate, diagnose and treat NP. Previous studies suggest that tailored exercise programs designed to address individual functional deficits may be more effective in reducing pain and disability than general physical activity or generic neck exercises [10]. However, these findings are not always consistent [11], and overall treatment effects tend to be small [12,13]. Importantly, treatment approaches focused on addressing specific neck deficits demand comprehensive and reliable assessment procedures across important functional domains [14]. To achieve optimal function, current clinical guidelines recommend evaluating motion limitations, sensorimotor impairments and other symptoms to classify people with NP into 4 categories: NP with mobility deficits, NP with movement coordination impairments and whiplash, cervicogenic headaches, or radicular NP [15]. Guidelines further endorse use of active treatments, including specialized exercise plans targeting the cervical and scapulothoracic regions [15,16]. Within these guidelines and also based on findings of Sterling et al [17] and Stenneberg et al [18], is an implicit assumption that biomechanical impairments are consistently related to the magnitude of experienced pain or disability. However, no prior systematic approach to confirm these assumptions has been identified.

While clinical evaluation of neck function has traditionally focused on range of motion (ROM), strength, and postural observations, recent decades have seen an expansion in the ways neck function is understood and evaluated. For example, In people with NP, alterations in cervical sensory inputs can lead to impaired sensorimotor control and disrupted cervical kinematics, and have shown potentially important associations with ratings of disability or interference with daily activity [14,18,19]. The proliferation of low-cost ‘wearable’ devices such as Inertial Motion Units IMUs) in smartphones [20] or watches [21] means that collection of advanced kinematic metrics once limited to lab-based settings can now be implemented in routine clinical practice, though the relative value of such metrics or their accuracy remains largely unclear [22].

Kinematics refers to the motion of objects in space by tracking their position vectors, such as how the head moves in relation to the torso [23]. Accessible motion tracking technologies have enabled high-precision kinematic analyses in NP that go beyond single-plane ROM metrics. For instance, recent research has explored time derivatives of the position vector that are dynamic and arguably closer to ‘real-world’ neck function [24–27]. This includes examining motion parameters like velocity (the first derivative), acceleration (the second derivative), and jerk (the third derivative). These parameters provide objective metrics of how the head orients its sensory organs toward environmental stimuli and have been studied to gain a deeper understanding of the quality and precision of neck movement [19,27–29] that may be related to patient-reported pain and disability.

While clinical biomechanical data provide some information about the state of neck function, tenets of patient-centred care place patient experience at the centre of clinical practice and research [30]. Accordingly, patient reported outcome measures (PROMs) have become widely accepted as a standard approach to capturing and quantifying patients’ subjective experiences of pain or disability [31]. In NP, the most common PROMs used to capture patient experiences are pain intensity (e.g., Numeric Pain Rating Scale [32]) and pain-related disability (e.g., Neck Disability Index [33,34]). Neither PROMs nor clinical biomechanics offer a complete view in isolation, and many authors endorse integration of data from across different assessment approaches for a more holistic understanding [35,36].

## Objectives

To systematically search and synthesize existing evidence on the association between clinical biomechanical metrics of neck function (e.g., ROM, strength, acceleration, accuracy, smoothness, etc.) and subjective reports of pain and disability amongst adults with neuromusculoskeletal NP. In this review, the focus will be on the biomechanical metrics of the cervical spine that are feasibly measurable in a typical rehabilitation clinical setting (further defined below).

## Methods and analysis

### Design

This systematic review protocol is designed using the Preferred Reporting Items for Systematic Review and Meta-Analysis Protocols statement (S1 File) [37] and is aligned with guidelines in the Cochrane Handbook [38]. The protocol is registered with PROSPERO (registration number CRD42023417317). Ethical approval was not sought for this study, as it involves the synthesis and analysis of existing literature.

### Eligibility criteria

Studies will be included according to the criteria outlined below informed by an adapted PICOS (Participants, Intervention, Comparator, Outcome, and Study Design) framework [39–41].

#### Participants

Studies investigating adults (18 years or older) with acute, subacute, or chronic NP with or without radiation will be included. This review focuses on people with neuromusculoskeletal neck pain. Studies focusing on pain arising from neck fractures or dislocations, myelopathy, cancer or tumor, autoimmune disease and infection, systemic inflammation, and post-surgical studies will be excluded.

#### Intervention and comparator

Neither comparator nor intervention are considered for this review.

#### Outcome

The studies must report and evaluate the association between at least one clinical biomechanical metric of neck function and one standardized and validated patient-reported outcome related to pain or disability of the neck.

#### Clinical biomechanical metrics

The term biomechanics refers to any motion-based quantification of neck function performed under active (volitional) control of the patient/participant. To ensure the findings are relevant to rehabilitation clinicians and researchers, “clinical biomechanics” is defined as quantitation of volitional movement-based parameters that can be feasibly observed or captured by a third-person evaluator in a rehabilitation clinical environment. Common clinical biomechanical metrics will be range of motion (single or multiple planes), strength (static or dynamic), or endurance of the neck muscles [19,29,42]. While not yet routine, the emergence of accessible IMUs means that more advanced metrics like accuracy/proprioception, velocity/acceleration, and smoothness/jerk can also be considered feasible in a standard rehabilitation setting although accuracy and reliability of such data for neck evaluation has yet to be confirmed [43,44]. Not included will be biomechanical metrics that require advanced techniques such as real-time or dynamic imaging (e.g., fluoroscopy), cadaveric dissection, or implantable sensors.

#### Patient reported outcome measures

PROMs are carefully developed, validated and standardized tools such as questionnaires to capture any report coming directly from the person experiencing NP related to their experiences with pain, functional impairment, or well-being [45]. This review will focus on PROMs measuring experiences of pain and disability that have been adequately validated by at least one prior study for use in people with neuromusculoskeletal NP [46,47]. These may be specifically related to the neck (e.g., Neck Disability Index [33,34]) or more generic (e.g., Pain Disability Index [48]). Pain scales are expected to most commonly be captured using the Visual Analog Scale (VAS) [49] or Numeric Pain Rating Scale (NPRS) [32]; however, more complex pain scales will be included if appropriate (e.g., Brief Pain Inventory [50], P4 Pain Measure [51], McGill Pain Questionnaire [52], etc.)

#### Study design, language, publication, setting, and time frame

Observational studies, including retrospective and prospective longitudinal cohort, and cross-sectional studies are included if they have presented a cross-sectional (collected at the same time) [53] analysis of the association between at least one clinical biomechanical metric of the cervical spine and at least one adequately valid PROM in people with neuromusculoskeletal NP.

To reduce the risk of bias, studies of all languages will be included in the search. However, due to limitations in time and resources, studies not in English or that do not have a professional translation into English already available will be excluded but noted on the PRISMA flow diagram. No limitation on publication date or location will be applied. There will be no restrictions by type of setting, though this will be captured as part of the data extraction process.

### Information sources

A systematic search in the following electronic databases will be conducted from inception to 20 November 2023: MEDLINE, Embase, Scopus, Web of Science, SPORTDiscus, and the Cumulative Index of Nursing and Applied Health Literature (CINAHL). No restriction will be imposed on the region of the studies. Literature search will be limited to human subjects.

The grey literature will include conference abstracts/proceedings and dissertations found in Embase and Scopus. The ProQuest Dissertations & Theses Global (PQDT) will be searched for relevant dissertations and theses (at https://www.proquest.com/pqdtglobal/advanced). Other sources of health-related grey literature (e.g., “Grey Matters” (at https://greymatters.cadth.ca/) will be searched to ensure saturation of knowledge. The reference lists of included articles and other relevant reviews will be hand-searched to identify additional articles for inclusion.

### Search strategy

The search strategy was developed in MEDLINE Ovid (S2 File) by the lead author in consultation with the research team who hold expertise in NP and systematic reviews, and an experienced research librarian using the Peer Review of Electronic Search Strategies (PRESS) standard [54]. The search strategy consisting of medical subject heading (MeSH) terms and text words was subsequently tailored for the other databases. The search terms mainly include the two groups of the outcomes measured by PROMs (such as neck pain, neck injuries, disability) and clinical biomechanical measures of cervical spine (such as ROM, kinematics, biomechanics, movement).

### Study records

#### Data management

The results of the literature search and citations will be imported and sorted in Covidence, a web-based software tool for conducting systematic reviews [55,56]. Duplicate studies will be identified and removed by this software prior to the screening process. Full text of potentially eligible studies identified after the title and abstract screening will be uploaded and stored in Covidence where both stages of screening occur.

#### Selection process

**Fig 1:**
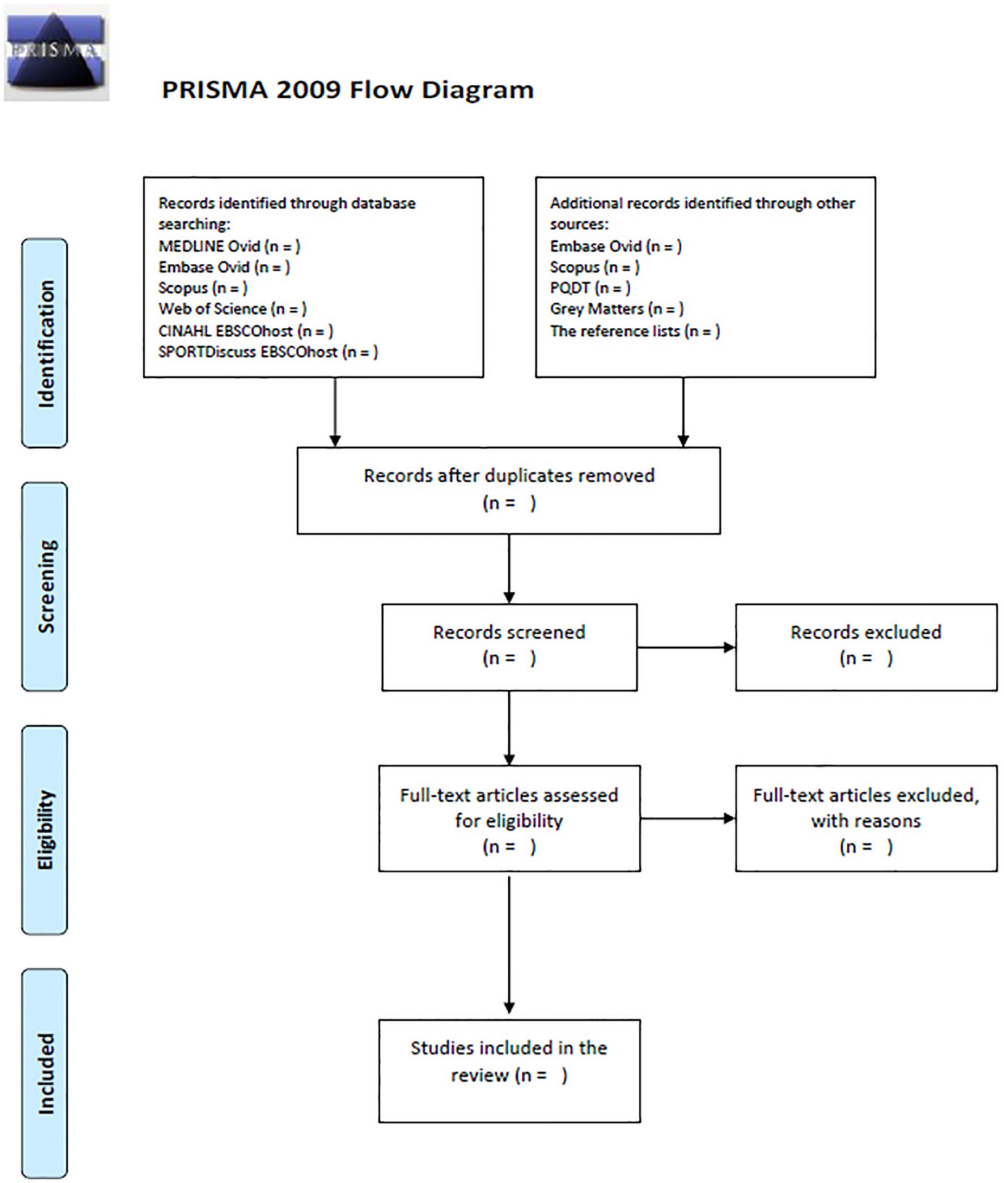
Flow diagram of study selection processes. *From*: Moher D, Liberati A, Tetzlaff J, Altman DG, The PRISMA Group (2009). *P*referred *R*eporting *I*tems for Systematic Reviews and *M*eta-*A*nalyses: The PRISMA Statement. PLoS Med 6(7): e1000097. doi: 10.1371/journal.pmed.1000097 For more information, visit www.prisma-statement.org.

The eligibility assessment will be performed independently by two reviewers (SS, SV) starting with title and abstract screen to remove clearly irrelevant studies. In the second stage, full texts will be independently screened for inclusion, with agreement from both reviewers required based on the predefined criteria. Disagreements at both stages will be discussed and if consensus is not achieved, a third reviewer (DW) will mediate. Cohen’s kappa will evaluate inter-reviewer agreement in both stages [57]. The entire selection process along with exclusion reasons will be documented in the PRISMA flow diagram (Fig 1) for transparency and clarity [58].

#### Data collection process

Two reviewers (SS, SV) will perform data extraction independently using standardized data extraction forms. The forms will be piloted with five articles and modified where needed to ensure adequacy and between-reviewer calibration. Any disagreements in the data extracted will be discussed and resolved between the review team. Where important data are unclear or missing, the corresponding author of the manuscript will be contacted via email. Where the author does not respond after two follow up reminder emails within two-week intervals, only available data will be extracted.

### Data items

Data items to be extracted from the included studies are shown in Table 1.

**Table 1:** Summary of data to be extracted from included studies.

Study characteristics (background and methodology)	Title, Authors, year of publication, study design, setting, country of study, objective(s) of the study
Participants, demographics, and clinical characteristics across included studies	Age, sex (proportion), sample size (per group if more than one), inclusion and exclusion criteria, Author’s definition of cases and their classification of neck pain (including its severity and duration where available), Author’s definition of controls (if any), time point post injury (if reported), participants history of neck pain/injury and other health conditions (if available)
Outcome measures and methods of outcome measurement	1. Clinical biomechanical measures of head and neck movement (2D and 3D), tools used, and number of assessments, mean values. 2. PROMs: Name, version and/or subscale, method of administration, number of assessments, mean values. 3. Results and author interpretations of the association between 1 &2

### Outcomes and prioritization

The main outcome measures for this study, as mentioned previously under the eligibility criteria heading on pages 7 and 8 are in short 1) Clinical biomechanical measures of neck, and 2) self-reported measures.

### Risk of Bias (RoB) for individual studies

The U.S. National Institutes of Health (NIH) Quality Assessment Tool for Observational, Cohort, and Cross-Sectional Studies (QATOCCS [59]) will be used to assess the risk of bias (RoB) in the included studies, and has been shown to be valid and reliable for observational cohort and cross-sectional studies [59,60]. As mentioned in the inclusion criteria for this review, observational cohort and cross-sectional designs that present a concurrent association between at least one PROM and one clinical biomechanics metric will be included. A RoB tool that can be applied to different study designs is therefore necessary, and the QATOCCS satisfies that criterion [19,29]. It consists of fourteen items assessing the objectives, study population, the measurement of the exposure and condition, the presence of confounding factors, and the statistical analyses used, with ‘Yes’, ‘No’, ‘Other (Cannot Determine or Not Applicable or Not Reported)’ response options [59]. Overall, the studies will be rated as “poor”, “fair”, and “good” based on the number of questions that were answered yes out of 14 questions, respectively 0–4, 5–10, and 11–14. As explained by the NIH, High risk of bias translates to a rating of poor quality, fair are studies susceptible to some bias but not enough to invalidate its results, and low risk of bias translates to a rating of “good” quality [59].

Two independent reviewers (SS, SV) will assess RoB using the QATOCCS. In case of disagreements, they will discuss to reach a consensus, and if they are unable to resolve the conflicting views, a third reviewer (DW) will mediate. Cohen’s kappa will be used to assess the agreement between reviewers and reported [57]. All tools and processes will be piloted prior to use on a randomly selected subset of 2–3 papers to ensure calibration of reviewers before independent scoring.

### Data synthesis

If studies are sufficiently homogenous based on the design, population, and outcome measures (clinical biomechanical measures and PROMs) and if adequate data are available meta-analyses (MA) will be conducted. However, based on an initial scoping search, it is expected that clinical heterogeneity will be evident across samples with respect to measures, study design, and characteristics of NP that may preclude statistical synthesis. Accordingly, the project will be initiated with the goal of conducting a meta-analysis, while also describing a narrative review that will be decided upon once data are extracted.

#### Meta-analysis

Where appropriate, a random-effects MA will be performed with the pooled effect indicator most likely to be Pearson’s r with 95% confidence intervals. Spearman’s rho, r-squared, or regression coefficients may be usable to estimate the correlation coefficient in the MA if sufficient supplementary data are available (e.g., unstandardized univariate effects are presented in studies using multivariate regression). I^2^ test and Q values will be used as indicators of effect homogeneity. Where significant heterogeneity exists in effect sizes, potential explanations will be sought through moderator analysis, with effects stratified by meaningful variables, including the duration of symptoms or type of NP.

#### Proposed additional analyses

Based on the classification of NP proposed by current clinical guidelines [15] an attempt will be made to analyze the data within the recommended subgroups of: 1) NP with mobility deficits, 2) NP with movement coordination impairments and whiplash, 3) NP with headaches, and 4) NP with radicular pain. Other subgroup analyses could be based on the duration and chronicity of NP in subgroups of: 1) acute NP, 2) subacute NP, and 3) chronic NP [61]; provided an adequate number of studies possessing all the characteristics described are included. Other potential additional analysis such as subgrouping based on different outcomes or outcome measures will be decided upon in case of sufficiently homogeneous studies.

#### Narrative synthesis

Where MA cannot be conducted due to heterogeneity, the results will be summarized in a narrative format in accordance with guidance by the Cochrane Consumers and Communication Review Group [62], and using the adapted GRADE approach (Grading of Recommendations Assessment, Development and Evaluation) for reviews of observational or prognostic factors [63] to rate the overall confidence in the level of association based on RoB, inconsistency, indirectness, imprecision, and publication bias.

The guidelines describe four major steps for narrative synthesis. The first step of ‘developing a theory of how the intervention works’ will be omitted as this review does not involve interventions. This aligns with approaches adopted in previous studies [64]. The findings of eligible studies will be extracted, summarized, and organized based on similarities of clinical biomechanics in tabular format to identify patterns and relationships in the data across the included studies. Results will first be grouped by clinical biomechanical metric (e.g., ROM, accuracy) and by PROM construct (pain or disability) with synthesis occurring for each metric by each PROM construct. Magnitude of association (e.g., correlation or variance explained) is the primary construct of interest. While interpretation of correlation coefficients differs significantly among scientific research areas and there are no absolute rules for the interpretation of their strength [65], it is possible to categorize the strength of these relationships as small (0.10–0.29), medium (0.30–0.49) and large (≥0.50) [66].

#### Meta-bias (es)

It is recommended by PRISMA [39] that that systematic reviews explore reporting bias of included studies. Considering the inclusion criteria, which focus on observational study designs, it cannot be presumed that the protocols of these studies were registered before publication. Consequently, determining reporting bias might be challenging. Publication bias will be explored in the narrative synthesis following the GRADE approach [67], or if MA has been conducted Rosenthal’s fail-safe number or *fail-safe N* [68] can be calculated to estimate the “file-drawer effect”.

### Confidence of cumulative evidence

If a meta-analysis is not possible, a narrative synthesis will be conducted following the Grading of Recommendations Assessment, Development and Evaluation (GRADE) working group methodology to provide information not only on the internal validity (risk of bias) of all included studies, but also external validity (inconsistency, indirectness, etc.) [69]. GRADE will be used to rate the overall quality of evidence based on five criteria including the RoB (QATOCCS [69]), consistency of results across studies, relevance of the study design to our population(s) of interest (e.g., adults with neuromusculoskeletal neck pain in rehabilitation settings), imprecision (e.g., wide confidence intervals around point estimates) and publication bias. The certainty of evidence will be rated as high, moderate, low, and very low [69] using the adapted GRADE approach for the assessment of evidence of observational and prognostic factors [63,70]. Final decisions on evidence summaries will be team-based.

According to the GRADE guidelines and the Cochrane Handbook [38], observational studies will initially be given a ‘low’ rating for the quality of evidence. Then the quality of evidence can be either upgraded or downgraded from this point [71]. Studies will be upgraded for factors such as 1) large effect sizes, 2) confidence in the accuracy of findings that consider the risk of bias, directness of study design, consistencies between studies in terms of populations studied, measures used, and the extent to which the sample studied is representative of the desired population [69,71]. Studies could be downgraded in the case of high RoB, inconsistent results, indirectness of evidence, imprecision, or suspicion of publication bias (missing data, selective reporting, or clear differences between study registration and published manuscript) [42].

## Discussion

NP is a highly prevalent condition that can have a significant impact on a person’s work, recreation, and quality of life. According to findings of the Global Burden of Disease Study (GBD) 2021 [72], NP has been one of the leading causes of years lived with disability since 1990. Although neck pain could have various origins, musculoskeletal causes are by far the most common [14] which are the focus of this study.

Pain and injury to any region of the musculoskeletal system, including the cervical spine, can have profound effects on the neuromuscular system that may be related to the experience of neck pain and disability. Prior work has shown kinematic impairments in some patients with NP [19,26,28,73,74], however it is not yet clear what specific impairments are most strongly associated with pain and disability.

This systematic review will provide a comprehensive assessment of current evidence. The results are expected to benefit (1) health practitioners in selection of exercises, rehabilitation methods and protocols, or suitable and efficient assessment tools based on the clinical biomechanical patterns most associated with NP; (2) patients who can use the information to seek the most relevant healthcare professional(s) for managing their neck pain; and (3) researchers and policy makers who can use the strongly associated clinical biomechanical metrics of neck motion in designing research rials and rehabilitation protocols which in turn will improve health assessment and patient care.

## Supporting information

S1 File PRISMA-P checklist. (DOCX)

S2 File Search strategy in the MEDLINE Ovid. (DOCX)
