# Supplementary material for: Association between clinical biomechanical metrics of cervical spine function and pain or disability in people with neuromusculoskeletal neck pain: Protocol for a systematic review and planned meta-analysis
Source: PLoS One. 2024 May 10;19(5):e0303365. doi: 10.1371/journal.pone.0303365 (PMC11086898; doi:10.1371/journal.pone.0303365)
Supplement: S2 File — (DOCX) [file pone.0303365.s002.docx]

# Ovid MEDLINE(R) Search Strategy

1 Neck Pain/

2 neck injuries/ or whiplash injuries/ or ((neck or cervic*) adj4 injur*).tw,kf.

3 ((neck or cervic*) adj4 (pain* or ache* or disabil*)).tw,kf.

4 (cervicalgia or Cervicogenic or cervicocephalic).tw,kf.

5 ((Radicul* or radiat*) adj5 (neck or cervic*)).tw,kf.

6 whiplash.tw,kf.

7 Radiculopathy/

8 Temporomandibular Joint Disorders/ or (temporomandibular adj4 (disorder* or disease* or dysfunction* or syndrome*)).tw,kf.

9 Biomechanical Phenomena/

10 Kinesthesis/

11 "Range of Motion, Articular"/

12 Movement/

13 Movement Disorders/

14 Proprioception/

15 ((biomechanic* or dynamic* or kinematic*) adj5 (head or neck or cervical)).tw,kf.

16 ((movement* or motion* or mobility* or motor*) adj5 (head or neck or cervical)).tw,kf.

17 ((position* or accuracy or velocity or accelerat* or jerk* or smooth*) adj5 (head or neck or cervical)).tw,kf.

18 (function adj5 (head or neck or cervical)).tw,kf.

19 ((strength or endurance) adj9 (head or neck or cervical)).tw,kf.

20 propriocept*.tw,kf.

21 (Sensorimotor or sensori-motor).tw,kf.

22 kinesth*.tw,kf.

23 1 or 2 or 3 or 4 or 5 or 6 or 7 or 8

24 9 or 10 or 11 or 12 or 13 or 14 or 15 or 16 or 17 or 18 or 19 or 20 or 21 or 22

25 23 and 24
